# Community-Based Organizations’ Approaches to Recruitment and Retention for a Digital HIV Prevention Intervention for Young Men Who Have Sex With Men: A Mixed Methods Study

**DOI:** 10.2196/63199

**Published:** 2025-11-03

**Authors:** Alithia Zamantakis, Elizabeth Danielson, Emma Rudd, J Pablo Zapata, Nanette Benbow, Rana Saber, Ashley A Knapp, Brian Mustanski

**Affiliations:** 1 Impact Institute, Northwestern University, 625 N. Michigan Avenue, Suite 1400, Chicago, IL, 60611, United States, 1 3125035408; 2 Medical Social Sciences, Northwestern University, Chicago, IL, United States; 3 Center for Dissemination and Implementation Science, Northwestern University, Chicago, IL, United States; 4 Department of Psychiatry and Behavioral Sciences, Northwestern University, Chicago, IL, United States

**Keywords:** YMSM, community-based organizations, recruitment, retention, HIV prevention, digital health intervention, men who have sex with men, risk reduction, sexual health, reproductive health, health disparities, sexually transmitted infection

## Abstract

**Background:**

Digital health interventions (DHIs) can broaden the reach of HIV prevention interventions and overcome barriers for young cisgender men who have sex with men (YMSM). Community-based organizations (CBOs) have delivered HIV prevention interventions for decades, but few studies have examined how CBOs implement DHIs, including recruitment and retention. Keep It Up! (KIU!) is a Centers for Disease Control and Prevention–designated best-evidence DHI that can promote risk reduction behaviors and reduce sexually transmitted infection incidence.

**Objective:**

We sought to descriptively assess CBOs’ approaches to recruitment and retention, elucidate lessons learned, and provide examples of recruitment and retention for future implementers.

**Methods:**

Twenty-two CBOs in counties with high HIV rates and large proportions of YMSM were selected through a request for proposal process to implement KIU!. Data were extracted from request for proposal applications and notes from monthly calls with CBO staff. Twenty-five staff members across CBOs were interviewed in the middle of implementation. A descriptive thematic analysis of the lessons learned in recruiting and retaining participants was performed. The research team developed an application dashboard for CBO staff to register participants, track participant progress through the intervention, record and track participant contact, and export usage data. CBO logins to the KIU! dashboard were tracked across the implementation. To descriptively compare approaches to recruitment and retention, the study team divided CBOs according to annual HIV testing volume in the 3 years prior to implementation and years of HIV service provision to YMSM.

**Results:**

The most frequent modes of recruitment were outreach and community partnerships (21/22, 95%), and the least frequent modes were via hook-up apps (6/22, 27%) and participant referrals (5/22, 23%). CBOs with a low HIV testing volume used online recruitment slightly more frequently, while medium-volume CBOs most frequently used hook-up apps for recruitment. Low-volume CBOs more frequently used phone calls and emails to remind participants to complete intervention modules, while high- and medium-volume CBOs more frequently used text messages. CBOs with more years of HIV service provision to YMSM more frequently had a set reminder schedule for contact with participants. CBO staff identified a need to change how KIU! is pitched to clients by using personalized, recipient-centered language rather than technical jargon. CBOs changed intake forms to ensure that staff remembered to offer KIU! to participants. CBOs had a difficult time retaining participants despite holding in-person events for enrolled participants (eg, raffles and trivia nights) and altering the frequency with which they reminded participants to complete modules.

**Conclusions:**

Although CBOs had experience in implementing other evidence-based interventions with YMSM, there was no consensus on successful recruitment and retention strategies for this population. We have presented approaches that future CBOs may use in their own implementation of KIU!.

## Introduction

Research funding in digital health interventions (DHIs) has increased over the last 2 decades, given their potential for widespread health promotion and disease prevention [1-3]. With expanded access to mobile phones and the internet, even among low-income populations, DHIs can broaden the reach of prevention and treatment programs and overcome barriers presented by traditional, in-person interventions [4]. While traditional face-to-face interventions have been used for HIV prevention, the advent of DHIs as a platform for promoting health behavior change opens new avenues for developing effective strategies to combat HIV transmission. Incorporating DHIs into existing HIV prevention services is promising, yet few DHIs for HIV prevention have been implemented at scale beyond research studies, and little is known about scaling up their effectiveness in real-world settings [2,5]. Scaling up their effectiveness in real-world settings requires an assessment of how to recruit and retain participants.

For nearly 40 years, community-based organizations (CBOs) have been funded by the Centers for Disease Control and Prevention (CDC) to deliver HIV prevention interventions in affected communities primarily through face-to-face individual and small group interventions. Given the years of experience of CBOs, combined with their accessibility to the community, knowledge of the community, and credibility in the community, they are well-positioned to implement HIV DHIs. However, how CBOs implement HIV DHIs remains underdocumented. The primary barriers to successful implementation include limited organizational resources and a lack of adaptation guides [6,7]. Among the most significant challenges faced by CBOs in scaling HIV DHIs are recruitment and retention [8]. Numerous barriers prevent individuals from participating in HIV DHIs, including time constraints, technological challenges, and dissatisfaction with the perceived impersonal nature of technology [9,10].

To address these challenges, implementation science has increasingly called for enhanced dissemination strategies to improve recruitment and retention in HIV DHIs [11,12]. These strategies include diversifying how DHIs are marketed to consumers and incorporating co-design approaches to involve consumers in promoting DHIs [10]. Efforts to document and scale enrollment strategies have also intensified. These strategies include using text message reminders, encouraging consumers to create online accounts on platforms, or offering personalized assistance from health professionals or administrators [10]. While these dissemination and engagement methods require further evaluation, particularly within CBOs, financial incentives have consistently been a cornerstone strategy across studies and CBOs to facilitate recruitment, retention, and response rates [13-15]. Research in both HIV and other disciplines has demonstrated the effectiveness of financial incentives in improving these outcomes [10]. However, less is understood about how CBOs manage financial incentives. This includes how decisions are made to cap or increase financial incentives and how they are paired with other forms of incentives. Further research is needed to document these practices to better support the scale-up of HIV DHIs in real-world settings.

Pragmatic trials, such as the Keep It Up! (KIU!) trial, are intended to test the effectiveness of an intervention and its implementation under typical, real-world conditions. Pragmatic trials “emphasize a balance between internal and external validity” [16]. In other words, such trials emphasize a balance between the scientific rigor, generalizability, and implementation of an intervention in a way that reflects the real circumstances in which it would otherwise be implemented outside a trial (eg, staff or organizational capacity to make adaptations when needed [17]). As such, the results are more readily and realistically applicable to addressing health and health care issues as well as providing implementers with recommendations for future policy and practice [18-20]. This paper describes the various approaches CBOs used to recruit and retain KIU! participants and describes the lessons learned to inform future implementations of DHIs in CBO settings.

## Methods

### Study Data

The data for this study were obtained from a type III hybrid effectiveness-implementation trial [21], KIU!, which compared 2 delivery approaches: direct-to-consumer and CBO-based implementation. The study protocol has been discussed elsewhere [22]. This manuscript focuses on the CBO-based implementation approach to recruitment and retention.

### Intervention: KIU!

KIU! is a CDC-designated best-evidence HIV risk reduction DHI. Previous iterations of KIU! have been discussed elsewhere [23-26]. KIU! was designed with and for young cisgender men who have sex with men (YMSM) aged 18‐29 years, who recently tested HIV-negative. KIU! supports risk reduction and promotes protection practices to maintain a negative HIV status. KIU! includes 3 modules focused on different prevention and risk negotiation content informed by the information-motivation-behavioral (IMB) skills model of HIV risk behavior change [27-29]. These modules are followed by 2 “booster” sessions 3 and 6 weeks after completion of the main intervention sessions. Booster sessions include additional encouragement of regular HIV testing, condom use, and pre-exposure prophylaxis (PrEP) uptake, and information on negotiating condom use with long-term partners.

As the effectiveness of KIU! has been previously demonstrated on behavioral outcomes (eg, condom use and casual anal sex) and biomedical outcomes (eg, sexually transmitted infection [STI] incidence [23,25]), the main aim of KIU! 3.0 was to (1) compare the 2 implementation delivery strategies, (2) examine the effect of strategies and determinants on variability in implementation outcomes using a mixed-methods research approach, and (3) explore the sustainment of KIU! at the completion of the study. This study (KIU! 3.0) was implemented in 22 CBOs located in counties with high HIV rates and large populations of YMSM.

### Participants

Twenty-two CBOs were selected through a request for proposal process similar to that used by HIV-prevention funders. Thirteen CBOs launched implementation in the fall of 2019, 7 in the summer of 2020, and 2 in the fall of 2020. KIU! 3.0 staff provided virtual training to CBOs on the intervention, provided capacity building support on how best to integrate it into routine HIV testing, and made monthly technical assistance calls. As part of the training, CBO staff completed the KIU! intervention to best describe it to clients during recruitment. CBO staff also received information about project logistics (ie, an online administrative dashboard to track recruitment and retention and manage project reporting via REDCap) and had time to ask any questions related to implementation within their organizations. While all CBOs recruited participants for the same intervention, how participants were recruited and retained varied across CBOs, as no set recruitment or retention plan was provided to them. CBOs were able to decide how to spend their implementation funding, how many staff members to include in the implementation, and whether to provide incentives to YMSM participants.

### Data Collection

This paper provides a comprehensive discussion and outline of the diverse range of activities that CBOs undertook while implementing KIU!. Furthermore, the manuscript details insights and lessons learned to enhance future implementation. Data for this paper were collected in several ways from CBOs: applications in response to a request for proposals, monthly standing calls, interviews, and the KIU! 3.0 application dashboard (Table 1). Data collection and analyses are reported in line with the Standards for Reporting Qualitative Research (SRQR; Checklist 1) [30]. While this manuscript uses quantitative data, the entire analysis is qualitative (ie, categorical) and descriptive.

**Table 1.** Data types and sources for this study.

Source	Data
Extracted from a request for proposals and notes from monthly calls^a^	Staffing logistics as pertains to KIU!^b^ Number of YMSM^c^ tested (“clients served”) in 2018 When and how KIU! is offered What types of promotional materials were developed by the CBOs^d^ (if any) Method of and schedule for communication with registered participants Participant events On-site accommodations (eg, space to complete KIU! at the CBO) Incentives given
Application dashboard	Number of times CBO staff logged into the dashboard
Interviews^e^	Lessons learned (ie, what they would do differently if they were to implement again)

a These data included what was proposed in CBOs’ responses to a request for funding proposals to implement KIU!, a digital health intervention for YMSM. Funding proposals were submitted between March and September 2019 by CBOs in 66 randomly selected US counties with large populations of YMSM. These data also include what actually took place during the 2 years of implementation, as collected from notes taken during monthly meetings with CBO staff and administrators. Monthly calls took place between December 2020 and April 2022, with a range of CBO staff involved in implementation from each CBO. To validate the data extracted from funding proposals and monthly meeting notes, we performed member checking with CBO staff in the middle of implementation.

b KIU!: Keep It Up!.

c YMSM: young cisgender men who have sex with men.

d CBO: community-based organization.

e In-depth interviews took place with staff at the 22 CBOs implementing KIU! (during implementation). Interviews were conducted between March 2021 and February 2022 on Zoom. No compensation was provided for staff members to participate in interviews.

#### Request for Proposals and Monthly Standing Calls

Data were extracted from responses to the request for proposals submitted between March and September 2019 and meeting notes from standing monthly calls between December 2020 and April 2022, with a range of staff involved in implementation from each CBO. Notes from standing monthly calls were collected by a research assistant using a note template capturing recruitment and retention successes, challenges, and plans for improvement. The data extracted from the request for proposals and monthly meeting notes were entered into an Excel spreadsheet (Microsoft Corp) and separated according to recruitment-related activities (eg, offering KIU! during HIV testing) and retention-related activities (eg, frequency of communication with participants). To validate the data extracted from the request for proposals and monthly meeting notes, member checking was performed with CBO staff in the middle of implementation.

#### Interviews

KIU! research staff trained in qualitative methods (including AZ and ER) aimed to conduct semistructured, in-depth interviews with 2 staff from each CBO who were designated by the CBO as implementers of KIU! in the middle of implementation. A total of 36 staff members were involved in the implementation of KIU! across the 22 CBOs (range=1‐3 per CBO). CBOs were each asked to select 2 staff members to participate in the interviews. Interviews in the middle of implementation were conducted between March 2021 and February 2022. Only 1 staff member participated in interviews from 9 CBOs, and 5 CBOs did not participate, resulting in a total of 25 interviews (response rate=56.8%). In the 5 CBOs that did not participate, the implementation staff did not consent to an interview. CBOs with only 1 staff member participating had a second staff member who did not consent to participate. Implementer roles varied across CBOs, with some staff holding managerial positions, others holding HIV testing positions, and others holding communications positions.

Interviews were based on the Consolidated Framework for Implementation Research (CFIR) [31]. Interviews occurred over Zoom (Zoom Communications), a videoconferencing software, and lasted an average of 1 hour and 15 minutes (range: 54 minutes to 2 hours). The interviews were recorded and automatically transcribed by Zoom. Research staff and senior investigators edited transcriptions to ensure consistency with the audio recordings, deidentified transcripts, and uploaded transcripts to MaxQDA (VERBI Software) for qualitative coding. Two of the authors with expertise in qualitative research conducted a descriptive thematic analysis of CBO staff responses using a constant comparison method [32]. For this study, we focused on staff responses to the question, “Based on your experiences delivering KIU!, if you could do things differently to improve any aspects of the implementation, what would they be?” This analysis focused only on responses related to probes regarding recruitment and retention. These data have been used to highlight CBO recommendations for future implementations of KIU! and other DHIs.

#### Administrative Dashboard

The KIU! application dashboard was developed to allow CBO staff to register participants, track their progress through the KIU! intervention, record and track participant contacts, and export usage data related to the participants. The KIU! dashboard also provided CBO staff with task lists to allow the easy identification of participants who needed additional reminders to complete the KIU! content. The KIU! study team tracked the number of logins into the dashboard per CBO during KIU! implementation (January 2020-April 2022) to assess the extent to which CBOs used the dashboard to monitor participant retention and intervention completion.

### Analysis

Client volume was divided (ie, annual HIV testing volume) roughly into thirds. CBOs having an annual HIV testing volume of YMSM between 20 and 139 were categorized as low volume. Those with a YMSM HIV testing volume of 140‐339 were categorized as medium volume, and those with a YMSM HIV testing volume of 340 or more were categorized as high volume. Regarding years of HIV service provision, we divided CBOs into 3 categories based on decades of experience. Those with 3‐9 years of experience were categorized as low (n=3), those with 10‐19 years of experience as medium (n=5), and those with 20 or more years of experience as high (n=14). CBO characteristics were compared by client volume and years of HIV service provision.

The analysis plan was not prespecified as the study was not intended to include a statistical analysis detailing whether one recruitment and retention approach was superior or more effective than another. Instead, the aim was to document these various approaches and detail the lessons learned, as CBO recruitment and retention for DHIs are relatively new concepts. This manuscript thus provides a contextual description of CBO approaches to reach YMSM for an HIV-prevention DHI, as a means to disseminate this information for future CBOs implementing KIU! or other DHIs. A report involving complete statistical analysis of the effectiveness and implementation of KIU! is currently under review [33], and the research team has recently published a latent class analysis examining the differences in implementation outcomes across classes of substance abuse [34].

### Positionality

The research study team included 1 research study assistant, 2 investigators with master’s-level experience, and 4 investigators with doctoral-level experience. One investigator had over 10 years of experience working in a local health department (NB), and 2 were experts in qualitative research methods (AZ and JPZ). The research study team predominantly included white individuals and women.

### Ethical Considerations

The study protocol was approved by the institutional review board (IRB) at Northwestern University (IRB reference number: STU00207476) [22]. YMSM and CBO staff participants were informed of the aims of the study as well as data protection. All participants provided consent to participate in our trial. No compensation was provided to CBO staff for participating in interviews. Identifiable information about participating organizations has been removed.

## Results

### CBO Characteristics

The 22 participating CBOs were located within the South, West, Midwest, and Northeast regions of the United States, with 1 outside the continental United States. All CBOs provided HIV testing services and either directly offered or referred individuals to PrEP services. Among the YMSM who accessed HIV and STI services from these CBOs between 2016 and 2019, 43.0% (3336/7759) were white, 22.0% (1707/7759) Black, 10.0% (776/7759) Asian, 9.0% (698/7759) multiracial, and 8.0% (620/7759) American Indian/Alaskan Native. Moreover, 68.0% (5276/7759) of clients were non-Hispanic/Latino, and 32.0% (2483/7759) were Hispanic/Latino. CBOs tested an average of 352.68 YMSM in 2018 (median 241; range 20‐1025).

Of the 22 CBOs, 13 (59%) had prior experience implementing evidence-based interventions (EBIs) such as VOICES/VOCES [35], CLEAR [36], and MPowerment [37]. Moreover, 14 (64%) CBOs had served YMSM for more than 20 years, 5 (23%) had served YMSM for 11‐20 years, and 3 (14%) had served YMSM for 10 or fewer years. While not required to do so, all CBOs offered some form of incentive to participants (eg, cash incentives or free HIV/STI testing). Incentive timing varied by CBO, with 16 (73%) offering incentives at baseline, 18 (82%) offering incentives after all 3 main episodes, and 20 (91%) offering incentives after booster episodes (Multimedia Appendix 1). CBOs, on average, provided a greater proportion of the total intervention incentive after boosters (48.3% of total incentives offered to participants; US $35.91 out of US $72.27), compared with 15.3% (US $11.36 out of US $72.27) at baseline and 33.6% (US $25.00 out of US $72.27) after completion of main episodes. Two CBOs provided incentives for participant referrals. Over half of CBOs (13/22, 59%) had their project managers directly involved in the implementation of KIU!. Project managers were mid- and high-level leaders, including deputy directors, program directors, and project coordinators.

### Client Volume

Medium-volume CBOs had slightly more staff involved in KIU! delivery than high- and low-volume CBOs (2.29 vs 2.13 and 1.43, respectively) (Table 2). Medium-volume CBOs also reported higher rates of project manager involvement in KIU! than high- and low-volume CBOs (5/7, 71% vs 4/8, 50% and 4/7, 57%, respectively). Low-volume CBOs had never been directly funded by the CDC for HIV prevention, while nearly 40% of medium- and high-volume CBOs had been funded (3/7, 43% and 3/8, 38%, respectively). Medium- and high-volume CBOs also had more experience implementing CDC-designated EBIs (5/7, 71% and 7/8, 88%, respectively).

**Table 2.** Recruitment and retention approaches.

Variable	Overall (N=22)	Clients served annually^a^			Years of HIV service provision^b^			Previous experience implementing CDC^c^-designated EBIs^d^^,^^e^	
		Low (20‐139; n=7)	Medium (140‐339; n=7)	High (≥340; n=8)	Low (3‐9 years; n=3)	Medium (10‐19 years; n=5)	High (≥20 years; n=14)	Yes (n=13)	No (n=9)
Characteristics									
YMSM^f^ receiving HIV tests per year (2018), mean	352.7	82.1	238.1	689.6	145.3	338.4	402.2	490.5	153.7
Staff involved in KIU!^g^ implementation, mean	1.95	1.43	2.29	2.13	1.67	1.40	2.21	2.00	1.80
CBOs^h^ with a project manager directly involved in KIU! implementation, n (%)	13 (59)	4 (57)	5 (71)	4 (50)	2 (67)	2 (40)	9 (64)	8 (62)	4 (44)
Number of CBOs ever directly funded by CDC HIV prevention funding, n (%*)*	6 (27)	0 (0)	3 (43)	3 (38)	1 (33)	1 (20)	4 (29)	6 (46)	0 (0)
CBOs with prior experience implementing CDC-designated EBIs, n (%)	13 (59)	1 (14)	5 (71)	7 (88)	1 (33)	3 (60)	9 (64)	—^i^	—
Recruitment									
Mode of recruitment^j^									
Online recruitment, n (%)	9 (41)	4 (57)	3 (43)	2 (25)	2 (67)	4 (80)	3 (21)	8 (62)	3 (33)
Hook-up app recruitment, n (%)	6 (27)	1 (14)	5 (71)	0 (0)	0 (0)	3 (60)	3 (21)	2 (15)	3 (33)
Participant referrals, n (%)	5 (23)	2 (29)	1 (14)	2 (25)	0 (0)	1 (20)	4 (29)	3 (23)	2 (22)
Outreach and community partnership, n (%)	21 (95)	7 (100)	7 (100)	7 (88)	3 (100)	5 (100)	13 (93)	9 (69)	7 (78)
Developed promotional recruitment materials, n (%)	15 (68)	5 (71)	4 (57)	6 (75)	1 (33)	4 (80)	10 (71)	8 (62)	7 (78)
Retention^k^									
Types of communication reminders									
Phone calls, n (%)	15 (68)	6 (86)	4 (57)	5 (63)	2 (67)	5 (100)	8 (57)	7 (54)	5 (56)
Text messages, n (%)	15 (68)	4 (57)	5 (71)	6 (75)	2 (67)	5 (100)	8 (57)	7 (54)	2 (22)
Emails, n (%)	13 (59)	6 (86)	3 (43)	4 (50)	1 (33)	5 (100)	7 (50)	5 (38)	3 (33)
Social media, n (%)	2 (9)	1 (14)	1 (14)	0 (0)	2 (67)	0 (0)	0 (0)	4 (31)	1 (11)
Frequency of reminders									
No reminder schedule, n (%)	6 (27)	2 (29)	2 (29)	2 (25)	1 (33)	2 (40)	3 (21)	2 (15)	4 (44)
Infrequent reminders^l^, n (%)	8 (36)	2 (29)	4 (57)	2 (25)	1 (33)	3 (60)	4 (29)	6 (46)	1 (11)
Frequent reminders^m^, n (%)	8 (36)	3 (43)	1 (14)	4 (50)	1 (33)	0 (0)	7 (50)	3 (23)	4 (44)
Offers accommodation to complete KIU!^n^, n *(%*)	8 (36)	1 (14)	2 (29)	5 (63)	1 (33)	2 (40)	6 (43)	3 (23)	1 (11)
Dashboard logins per month^o^, mean	10.5	13.0	7.4	11.1	12.0	11.4	9.9	7.2	14.3

a Client volume was divided roughly into thirds, as there were no preset thresholds. Client volume was collected from funding proposals that the 22 CBOs submitted between March and September 2019 in response to a request for proposals to implement KIU!.

b Years of HIV service provision included the number of years that CBOs stated they had been providing HIV services to any population. This information was collected from the same funding proposals as the client volume data.

c CDC: Centers for Disease Control and Prevention.

d EBI: evidence-based intervention.

e CBOs were asked to state whether they had previous experience implementing CDC-designated evidence-based interventions in their funding proposals.

f YMSM: young cisgender men who have sex with men.

g KIU!: Keep It Up!.

h CBO: community-based organization.

i Not applicable.

j Mode of recruitment includes the methods CBOs used during implementation of KIU! to recruit YMSM. We categorized modes of recruitment by the mechanism CBOs used for recruitment (eg, online webpage or phone app). These modes of recruitment were reported by CBO staff to study team members during monthly meetings between December 2020 and April 2022.

k Retention refers to the activities and the frequency of activities employed by CBOs to retain YMSM who have enrolled in KIU!. This was reported by CBO staff to study team members during monthly meetings between December 2020 and April 2022.

l Infrequent refers to a monthly or less frequent reminder.

m Frequent refers to a weekly or biweekly reminder.

n Accommodation refers to having space and technology for participants to complete KIU! at the CBO.

o The dashboard was developed to allow CBO staff to register participants, track participant progress through the KIU! intervention, record and track participant contacts, and export usage data related to the participants. CBOs reported these data between January 2020 and April 2022.

### Years of HIV Service Provision

CBOs with more years of experience providing HIV services tested more YMSM for HIV on average per year, with high-experience CBOs testing an average of 402.2 YMSM per year for HIV compared to 338.4 YMSM in medium-experience CBOs and 145.3 YMSM in low-experience CBOs. High-experience CBOs also had slightly more staff involved in KIU! delivery than medium- and low-experience CBOs (2.21 vs 1.40 and 1.67, respectively). High- and low-experience CBOs’ project managers were more frequently engaged in KIU! (9/14, 64% and 2/3, 67%, respectively) than medium-experience CBOs (2/5, 40%). Medium-experience CBOs less frequently reported funding from the CDC for HIV prevention than high- and low-experience CBOs (1/5, 20% vs 4/14, 29% and 1/3, 33%, respectively). Finally, high- and medium-experience CBOs more frequently reported prior experience implementing CDC-designated EBIs (9/14, 64% and 3/5, 60%, respectively) than low-experience CBOs (1/3, 33%).

### Prior Experience Implementing CDC-Designated EBIs

CBOs with prior experience implementing CDC-designated EBIs reported, on average, a higher number of YMSM receiving HIV tests per year (490.5 YMSM) than CBOs without prior experience (153.7 YMSM). CBOs with prior experience implementing EBIs reported a slightly higher average of staff involved in KIU! than CBOs without prior experience (2 and 1.8, respectively). However, CBOs with prior experience implementing EBIs reported more often than those without prior experience that project managers were directly involved in implementation (8/13, 62% and 4/9, 44%, respectively).

### Recruitment Activities

To recruit for KIU!, CBOs used four recruitment methods: (1) online recruitment (eg, on their website or on social media); (2) recruitment on hook-up and dating apps (eg, Grindr, Scruff, and Jack’d); (3) recruitment through participant referrals; and (4) recruitment through outreach and community partnerships. The most frequent mode of recruitment was through outreach and community partnerships (21/22, 95%), and the least frequent recruitment occurred via hook-up apps and participant referrals (6/22, 27% and 5/22, 23%, respectively; Table 2). Nearly 70% (15/22, 68%) of CBOs additionally created their own promotional materials to recruit participants in addition to materials provided by the research team. We compared the frequency of engagement in these activities by client volume of the CBOs, years of HIV service provision, and prior experience implementing CDC-designated EBIs (Table 2). We discuss these differences in approaches to recruitment below.

#### Client Volume

Regardless of client volume, nearly all CBOs (21/22, 95%) used outreach and community partnerships to recruit participants for KIU! (Table 2). Low-volume CBOs used online recruitment slightly more frequently than medium- and high-volume CBOs (4/7, 57% vs 3/7, 43% and 2/8, 25%, respectively). Medium-volume CBOs most frequently used hook-up apps as a medium of recruitment (5/7, 71%), with no high-volume CBOs using hook-up apps for recruitment. Fewer than a third of the CBOs in each category of client volume used participant referrals for recruitment, with only 1 medium-volume CBO doing so. High- and low-volume CBOs more frequently developed their own promotional recruitment materials (6/8, 75% and 5/7, 71%, respectively) than medium-volume CBOs (4/7, 57%).

#### Years of HIV Service Provision

Across years providing HIV services, nearly all CBOs used outreach and community partnerships to recruit participants for KIU! (Table 2). Low- and medium-experience CBOs providing HIV services more frequently used online recruitment (eg, social media and websites; 2/3, 67% and 4/5, 80%, respectively) than high-experience CBOs (3/14, 21%). Medium-experience CBOs reported using hook-up apps for recruitment more frequently than high- and low-experience CBOs (3/5, 60% vs 3/14, 21% and 0/3, 0%, respectively). Few CBOs (5/22, 23%) used participant referrals as a method of recruitment, with no low-experience CBOs doing so.

#### Prior Experience Implementing CDC-Designated EBIs

CBOs with prior experience more frequently reported using online recruitment than CBOs without prior experience (8/13, 62% vs 3/9, 33%). However, slightly more CBOs without prior experience than those with prior experience reported recruiting through outreach and community partnerships (7/9, 78% vs 9/13, 69%). CBOs without prior experience implementing EBIs more frequently reported developing their own promotional recruitment materials than CBOs with prior experience (7/9, 78% vs 9/13, 69%). Regardless of prior experience, only 14% (3/22) to 32% (7/22) of CBOs recruited through participant referrals or hook-up apps.

### Retention Activities

To retain participants, CBOs used four communication methods: (1) phone calls; (2) text messages; (3) emails; and (4) social media. Communication methods were primarily used to send reminders to participants to complete KIU! modules. Communication frequency varied across CBOs from no set schedule to infrequent (ie, monthly or less) and frequent (ie, weekly or biweekly). Phone calls and text messages (14/22, 64% for both) were the most common methods of communication, while social media was less common (2/22, 9%). Moreover, 36% (8/22) of CBOs chose to offer in-house accommodation to complete KIU! modules (eg, space and technology). In addition to CBOs’ individual approaches to retention, they were also able to use an online dashboard developed by our research team to track participant completion of KIU! modules. On average, all CBOs logged into the KIU! dashboard 10.5 times per month. We have compared modes of retention and dashboard utilization by client volume, years of HIV service provision, and prior experience implementing CDC-designated EBIs (Table 2). We discuss these differences in approaches to retention below.

#### Client Volume

Low-volume CBOs used phone calls more frequently than high- and medium-volume CBOs (6/7, 86% vs 5/8, 63% and 4/7, 57%, respectively). Low-volume CBOs also used emails more frequently than high- and medium-volume CBOs (6/7, 86% vs 4/8, 50% and 3/7, 43%, respectively), while high- and medium-volume CBOs used text messages more frequently (6/8, 75% and 5/7, 71%, respectively) than low-volume CBOs (4/7, 57%). One medium-volume and 1 low-volume CBO used social media, while no high-volume CBOs did.

Furthermore, 27% (6/22) of all CBOs, regardless of client volume, had no reminder schedule. Medium-volume CBOs most frequently had an infrequent reminder schedule (4/7, 57%), while high- and low-volume CBOs most frequently had a frequent reminder schedule (4/8, 50% and 3/7, 43%, respectively). High-volume CBOs most frequently offered accommodation to participants (5/8, 63%), while only 1 low-volume CBO did so. Low-volume CBOs logged into the KIU! dashboard, on average, more frequently than high- and medium-volume CBOs (13.0 vs 11.1 and 7.4 times, respectively).

#### Years of HIV Service Provision

All medium-experience CBOs used phone calls, text messages, and emails to contact participants. Low-experience CBOs used emails the least frequently (1/3, 33%), but were the only CBOs to use social media (2/3, 67%). A third (1/3, 33%) of low-experience CBOs had either no reminder schedule or an infrequent or frequent one. Medium-experience CBOs most frequently had an infrequent schedule (3/5, 60%), while high-experience CBOs most frequently had a frequent reminder schedule (7/14, 50%). Only 1 low-experience CBO offered accommodation to participants, while 43% (6/14) of high-experience CBOs and 40% (2/5) of medium-experience CBOs did. Finally, low-experience CBOs logged into the KIU! dashboard, on average, slightly more times than medium- and high-experience CBOs (12.0 vs 11.4 and 9.9 times, respectively).

#### Prior Experience Implementing CDC-Designated EBIs

There was little difference in the number of CBOs with and without prior experience implementing EBIs that reported communicating reminders via phone calls (7/13, 54% and 5/9, 56%, respectively) and emails (5/13, 38% and 3/9, 33%, respectively). CBOs with prior experience more often reported using text messages (7/13, 54%) and social media (3/9, 33%) than those without prior experience. CBOs without prior experience more often reported a frequent reminder schedule (4/9, 44% vs 3/13, 23%) and no reminder schedule (4/9, 44% vs 2/13, 15%). CBOs with prior experience implementing EBIs more often reported an infrequent reminder schedule (6/13, 46% vs 1/9, 11%). Moreover, CBOs with prior experience more often reported providing accommodation to complete KIU! (3/13, 23% vs 1/9, 11%). CBOs without prior experience logged into the KIU! dashboard, on average, more times per month than CBOs with prior experience (14.3 vs 7.2 times).

### Lessons Learned

We previously described differences in recruitment and retention activities by CBO client volume, years of HIV service provision, and direct project manager involvement in implementation. In recruiting and retaining participants, CBOs highlighted numerous lessons learned regarding what they felt helped with recruitment and retention and what hampered recruitment and retention. We now describe these lessons learned. In Table 3, lessons learned are compared by years of HIV service provision and project manager involvement. No discernible differences in lessons learned by client volume were found, and thus, these categorizations are not included within the table.

**Table 3.** Lessons learned in recruiting and retaining young cisgender men who have sex with men for KIU!^a^^,^^b^

Theme	Years of HIV service provision^c^			Project manager involvement	
	High experience (≥20 years), n (%)	Medium experience (10‐19 years), n (%)	Low experience (3‐9 years), n (%)	Direct involvement, n (%)	No direct involvement, n(%)
Change how KIU! is pitched to clients (n=5)	1 (20)	3 (60)	1 (20)	4 (80)	1 (20)
Incorporate recruitment into existing testing and PrEP^d^ programs (n=2)	0 (0)	1 (50)	1 (50)	2 (100)	0 (0)
Change intake forms (n=3)	2 (67)	1 (33)	0 (0)	0 (0)	3 (100)
Increase staffing (n=3)	1 (33)	1 (33)	1 (33)	2 (67)	1 (33)
Changes to who is involved in recruitment (n=3)	1 (33)	2 (67)	0 (0)	0 (0)	3 (100)
Online recruitment is helpful (n=3)	2 (67)	1 (33)	0 (0)	2 (67)	1 (33)
Online recruitment did not work (n=2)	0 (0)	1 (50)	1 (50)	1 (50)	1 (50)
COVID limited or hindered recruitment (n=8)	4 (50)	2 (25)	2 (25)	6 (76)	2 (25)
Use of in-person events for retention (n=1)	1 (100)	0 (0)	0 (0)	1 (100)	0 (0)
Retention calls (n=1)	0 (0)	1 (50)	1 (50)	1 (50)	1 (50)

a KIU!: Keep It Up!.

b Data were collected from in-depth interviews with staff from community-based organizations (CBOs) implementing KIU! between January 2020 and April 2022. Interviews were conducted by study team members during implementation between March 2021 and February 2022.

c Years of HIV service provision included the number of years that CBOs stated they had been providing HIV services to any population. This information was collected from funding proposals that the 22 CBOs submitted between March and September 2019 in response to a request for proposals to implement KIU!.

d PrEP: pre-exposure prophylaxis.

#### Recruitment

In interviews, CBOs highlighted the need to change how they pitched KIU! to their clients. CBOs described the need to point out the benefits of the intervention to participants and their communities rather than simply describing what KIU! is. Additionally, CBOs noted the importance of reaffirming that clients were not being offered KIU! because staff perceived them to be lacking in efforts to prevent HIV and framing the KIU! pitch to reinforce what clients were already doing to protect their health (eg, HIV testing). Further, using personalized, recipient-centered language rather than technical jargon and including the pitch within a larger discussion of holistic health prevention seemed to increase client registration. The timing of the pitch also mattered, with 1 CBO noting that it was difficult to recruit clients to participate when the offer came before HIV test results were delivered. In such cases, clients were too focused on whether their HIV tests would be reactive for the offer to be effective. One CBO also tried to pitch KIU! outside the testing room while clients waited for their test. However, this did not prove effective. A staff member explained:

*We initially had me kind of sitting out in the lobby with a table and talking to the clients as they were leaving their appointments—that wasn’t very well received by clients, because the lobby was a public area. People could overhear them about their LGBT status, and [clients worried about] if there’s going to be a negative response, so we were able to work with our provider to figure out a flow to integrate me into the actual clinic room.*

Two other CBOs had not originally planned to incorporate recruitment into their testing and PrEP programming, but during the implementation, they began to incorporate it and identified this as a source of increased recruitment.

CBOs also made changes to their intake forms to boost recruitment. These changes included ensuring that KIU! was listed on forms to be offered to participants, as well as ensuring that intake forms identified whether clients were eligible to participate. Once intake forms were completed, 1 CBO had a staff member review the form and place a sticker on the form to let counseling staff know whether the client was eligible for recruitment:

*We have tablets here, [but] it wasn’t giving us, it wouldn’t give us their age. So that’s one of the main reasons why we started putting stickers on their intake forms, because, yeah, you will see the birthday, but I feel like it was almost like bypassed for some reason. [Now], it was already pre-filled, already calculated for you.*

Staffing was also of concern to CBO staff in terms of the capacity to recruit. Staff turnover resulted in 3 CBOs having to retrain and reorient new individuals to implementing KIU! and also resulted in a limited number of staff (often 1) focused on recruitment amidst other ongoing work. One interviewee explained:

*We had like a pause because on recruitment because the previous person that had my title, he left like in June [or] July, so there was like six months that there wasn’t recruitment.*

Another CBO had been distributing flyers to promote KIU!; however, the staff member tasked with implementation left, resulting in a temporary end to promotion. The interviewee noted:

*The person that was previously working on the KIU! program, they’re not an employee anymore, so they [the fliers] kind of went away as far as like the promotion material.*

Interviewees also highlighted the need for more than one person to be tasked with recruitment, regardless of turnover. For example, 1 CBO trained all testing staff and volunteers on how to pitch KIU! to boost reach. However, staff in lower-level positions found it difficult to ask or tell colleagues to do so, stating that this request should come from upper management. Additionally, staff needed resources to support recruitment. One CBO found it helpful to provide each staff member with a cellphone and a laptop to keep track of client eligibility and to promote KIU!.

CBO staff diverged in their reflections on whether online recruitment aided their efforts. Three interviewees found their use of social media and hook-up apps to provide fruitful results. Interestingly, 1 CBO described LinkedIn as an effective social media site for recruitment:

*On social media, the (CBO) has Facebook, Instagram and LinkedIn. But they have posted like just a few, like maybe three times, and LinkedIn proved to be pretty effective.*

Another CBO compared their recruitment on 2 hook-up apps (Grindr and Scruff) with word-of-mouth recruitment and found better results online. However, they found it difficult to post on Facebook for recruitment due to Facebook’s restriction on sexually explicit content, which was described as having a potentially homophobic bias. They explained:

*Facebook’s ad policy has been on all of my ads for being too sexually suggestive or being sexually explicit. It’s gotten, to the point where they’ll say like your clothes to skin ratio is off and it’s just like....And then I look at the photo and it’s just like two men hugging and it’s the arms and face and then this much or chest is showing [makes gesture with hand to signify a small amount].*

Three other CBOs, though, highlighted a lack of results from online recruitment. One CBO felt that the lack of results may be due to the types of pages individuals seek out on social media. The interviewee elaborated:

*I don’t know that it’s incredibly successful. I don’t think a lot of people will follow health care program.*

Others highlighted difficulties with online recruitment due to fraudulent attempts to participate in KIU! from individuals or fake accounts that were not eligible to participate. One interviewee said:

*Once you put something like that out on the internet, and you say that you’re offering money, there are a lot of bots and scammers out there that are going to be reaching out. So we kind of pulled back a little bit on that. And we didn’t get a lot of traction from it outside of you know, bots and scammers. So we’ve moved away from that.*

There were no notable differences between CBOs that found online recruitment to yield results and those that did not. Both groups of respondents included staff from CBOs with a high, medium, or low number of years providing HIV services; with a high, medium, or low client volume; and with or without direct project manager involvement.

Finally, nearly one-third of interviewees (8/22, 36%; representing 5/22, 23% of all CBOs) highlighted difficulties with recruitment due to the COVID-19 pandemic. The first cohort of CBOs implementing KIU! launched implementation right before the pandemic began. For these and subsequent cohorts, implementation occurred alongside stay-at-home requirements, self-distancing, mask requirements, and pandemic-related layoffs that made recruitment a challenge to simultaneously manage. One interviewee commented:

*Originally, we were planning to have an in-person photo shoot. We were going to go to some of the LGBTQ businesses, like the bars and restaurants, and do in-person promotion and because of COVID-19, that hasn’t happened, because everything got shut down right? So that had to be pending, and then things are starting to open up here in [city] again and we’re just starting to think of that. We can finally plan things.*

#### Retention

Few interviewees offered reflections on retention (n=4). Among these interviewees, lessons and concerns they pointed to included discussion of in-person events for enrolled participants, calling participants for retention purposes, and general difficulties with retention.

In addition to having events for recruitment (eg, trivia nights where they encouraged participation in KIU!), 1 CBO also held in-person events for enrolled participants. At these events, staff and participants would play games and discuss participant questions about KIU! modules, and raffle prizes would be given to those who continued to complete KIU! modules. While this was seen as successful by the CBO, some challenges with this approach to retention were highlighted, as 2 participants were uncomfortable with in-person events and were upset that they were invited to something that was not entirely virtual. The CBO also began organizing virtual events on Zoom (Zoom Communications):

*[Colleague] organizes an event on Zoom once a month centered around some sort of theme. It’s usually sexual health related, but I think last month was sexual health plus nutrition, and we put together a gift basket for the client. If you attend, you have a chance of winning a gift basket and [there are] drawings where we give people $25 gift cards.*

Two interviewees also reflected on the use of phone calls for retention. One interviewee noted the difficulty in contacting participants this way, saying:

*They don’t really respond to our contact messages or calls.*

The interviewee, along with another interviewee at a separate CBO, felt unsure of how to retain participants despite trying various modes of contact. Another CBO, though, developed a “retention plan” to track participants. The retention plan included a spreadsheet of participants, when they had last been active, when they had been contacted, and who had contacted them. The interviewee implemented this to “lighten the load for myself and kind of do a cross check.”

Of note, only 1 high-experience CBO staff member reflected on retention successes and difficulties. The other 4 interviewees were from medium- and low-experience CBOs. Interviewees who reflected on retention were fairly evenly split between those with direct project manager involvement and those without (see Table 3).

## Discussion

### Principal Findings

Despite the interest in and uptake of DHIs, little is known about how local organizations, such as CBOs, engage in recruitment and retention in real-world settings. We analyzed phone or Zoom call transcripts and interview data to capture CBO recruitment and retention approaches to KIU! implementation. We also used qualitative analyses to identify lessons learned by CBOs related to recruitment and retention. In future analyses, the research team plans to use coincidence analysis to identify configurations of recruitment and retention activities that promote greater recruitment.

Throughout the implementation of KIU!, CBOs used 4 approaches to recruit participants: outreach and community partnerships, online recruitment (eg, on their website or on social media), hook-up and dating apps, and participant referrals. Overall, CBOs most often used outreach through community partnerships and online recruitment. Most CBOs also developed their own promotional recruitment materials, especially CBOs with prior experience implementing CDC EBIs. During interviews, CBOs acknowledged that their recruitment efforts should have focused on communicating the benefits of KIU!, using recipient-centered language (ie, removing jargon), and emphasizing that KIU! was for anyone and not targeted for individuals deemed at higher risk. CBOs also noted how the recruitment location introduced barriers to discussions with participants (in a waiting room compared to a private room). CBO staff were able to recognize that individual values and preferences were barriers to recruitment efforts. As a result, CBOs had to adjust their approaches by creating customized information about KIU! to communicate the benefits and find safe locations for participants to honestly answer personal questions about themselves in order to enroll in the intervention. CBO recruitment reflections echo the importance of tailoring content for the target audience and considering the perspectives of clients in intervention delivery [38-40]. These findings stress the importance of researchers working closely with their participant audience to anticipate recruitment barriers and collaboratively create facilitators.

For retention, CBOs used a variety of approaches. CBOs called, sent text messages, and emailed participants to remind them about KIU!. Additionally, 1 CBO hosted in-person events with prizes to promote continued engagement; however, these were met with mixed reviews from participants who expected events to be hosted virtually since KIU! is a digital intervention. Overall, CBOs noted that all retention methods had limited success. While reminders represented the most common approach, CBOs may need to consider using other research retention methods such as providing incentives, reducing barriers (providing alternative data collection methods), tracing, and emphasizing participation benefits [41,42]. Further, CBOs may want to work closely with participants to understand which retention strategies are the most meaningful to them [40].

### Scaling Up DHIs (Challenges and Potential)

DHIs have the potential to enhance the quality of care and reduce health care costs. Despite evidence that DHIs are effective for improving HIV prevention outcomes [25,30,43], the requirements to effectively scale these programs and bring them to practice are not well-established. The pre-established infrastructure, workflow, and experience of CBOs can create challenges for the scaling up of DHIs. Previous research indicates that the main obstacles to the adoption and scaling of DHIs include a lack of technical support, resistance to change, and financial constraints. Many CBOs lack technical proficiency and experience with implementing EBIs. These barriers make training a necessary component of implementation but can be a burden for CBO staff. CBOs also experience high turnover rates of staff who are involved in the recruitment and retention of participants, which can result in delays and deprioritization of recruitment for interventions that require additional training and cause gaps in retention as new staff undergo the training process. Workflows and strategies for recruiting and retaining participants for testing or in-person intervention modalities differ greatly from the implementation of DHIs, which could promote CBOs’ resistance to change or adapt already existing processes. Some CBOs also lack experience in retaining participants for an extended period of time, which is necessary for completing the learning objectives of online HIV prevention programs (eg, KIU! requires a minimum participant retention period of 3 months). All these challenges can be overcome with a better understanding of the optimal approaches of retention and recruitment activities to achieve DHI implementation success.

Although CBOs have been working with YMSM for many years and have experience implementing other CDC-designated EBIs, there is no consensus on successful recruitment and retention strategies for this population. This suggests that there is a critical need for implementation researchers to identify the best practices to improve the reach and benefits of future EBI implementation.

### Limitations

This study described the approaches real-world CBOs use to recruit and retain participants in a DHI for HIV prevention among YMSM. Although lessons learned are reported through interviews with CBO staff about intervention success, this manuscript does not report recruitment or retention rates, as the evaluation in this study was not part of a prespecified data analysis and was not meant to be a statistical analysis. Instead, a descriptive evaluation of the different recruitment and retention approaches CBOs used to implement a novel DHI for HIV prevention is provided. The results represent staff perceptions of success with different methods and are not associated with participant engagement or retention outcomes. The recruitment and retention methods listed here may not be representative of CBOs that did not choose to be enrolled in this study. CBOs self-selected to offer KIU!, and they may have had more resources and interest to engage in research activities. While the research team aimed to interview 2 staff members at each CBO implementing KIU!, CBO staff were not required in their funding contracts to participate in these interviews, and the research team was not willing to coerce participation. This resulted in 9 CBOs only having 1 staff member who consented to participate and 5 CBOs not having any staff member who consented to participate. Further, the staff who engaged in interviews varied greatly in their position titles across CBOs, which may have resulted in varied perspectives on implementation. This limitation was reduced by requiring those participating in interviews to be directly involved with the implementation of KIU!. Additionally, the retention findings were limited by few interviewee reflections on the approaches in the sample. This study took place during the COVID-19 pandemic. As a result, some of the reflections from CBO staff included challenges unique to that time period and may not be as relevant to recruitment and retention practices during stable economic and epidemiologic periods.

### Conclusion

Limited research has described the recruitment and retention practices of CBOs, especially for HIV prevention DHIs. CBOs providing HIV prevention services often have more experience recruiting clients than retaining them, given the nature of point-in-time services such as HIV testing and condom distribution. While some CBOs have experience with behavioral interventions for HIV prevention, DHIs like KIU! rely on CBO staff to ensure ongoing participant engagement and retention, even though the intervention takes place outside the CBO’s physical premises. In this trial of 22 CBOs implementing a DHI for HIV prevention, only a few CBOs had prior experience with DHIs, highlighting the need for dedicated staff and resources to successfully implement such interventions. These resources should include insights and strategies for effective recruitment and retention, as presented in this manuscript.

## Supplementary material

10.2196/63199 Multimedia Appendix 1 Information regarding incentives given throughout Keep It Up! implementation by community-based organizations.

10.2196/63199 Checklist 1 SRQR checklist.

## Abbreviations

CBO: community-based organization
CDC: Centers for Disease Control and Prevention
DHI: digital health intervention
EBI: evidence-based intervention
IRB: institutional review board
KIU!: Keep It Up!
PrEP: pre-exposure prophylaxis
STI: sexually transmitted infection
YMSM: young cisgender men who have sex with men
