# Supplementary material for: Community-Based Organizations’ Approaches to Recruitment and Retention for a Digital HIV Prevention Intervention for Young Men Who Have Sex With Men: A Mixed Methods Study
Source: J Med Internet Res. 2025 Nov 3;27:e63199. doi: 10.2196/63199 (PMC12582555; doi:10.2196/63199)
Supplement: Multimedia Appendix 1 [file jmir-v27-e63199-s001.docx]

| **Multimedia Appendix 1.** Information regarding incentives given throughout Keep It Up! implementation by community-based organizations.^1^ | |
| --- | --- |
| *Percent Giving Incentives...* |  |
| ...At Baseline | 72.7% |
| ...At Main | 81.8% |
| ...After Boosters | 90.9% |
| *Distribution of Incentives* |  |
| % Offered at Baseline | 15.3% |
| % Offered at Main | 33.6% |
| % Offered after Boosters | 48.3% |
| *Amount of Incentives* |  |
| Average Amount at Baseline | $11.36 |
| Average Amount at Main | $25.00 |
| Average Amount after Boosters^2^ | $35.91 |

^1^Data come from monthly standing calls.

^2^Amounts of incentive given after boosters includes CBOs who gave partial incentive for booster 1 and the rest of the incentive for booster 2, as well as CBOs who gave one incentive for participants who completed both boosters.
